# Quality of life, anxiety, and depression improve at one-year after liver transplantation in patients with advanced liver disease

**DOI:** 10.3389/frtra.2024.1476952

**Published:** 2024-11-21

**Authors:** Rosana Cordoba-Alvarado, Valentina Romero-Fonnegra, Nicolas Cortes-Mejia, Diana Fernanda Bejarano-Ramirez, Valentina Maldonado-Hoyos, Sandra Janeth Sanchez-Garcia, Alonso Vera-Torres

**Affiliations:** ^1^ Clinical Psychology Section, Transplant Services, Fundacion Santa Fe de Bogotá, Bogotá, Colombia; ^2^ Transplant and Hepatobiliary Surgery Department, Fundación Santa Fe de Bogotá, Bogotá, Colombia; ^3^ Faculty of Psychology, Universidad de los Andes, Bogotá, Colombia; ^4^ Division of Anesthesiology Critical Care Medicine, and Pain Medicine, The University of Texas MD Anderson Cancer Center, Houston, TX, United States; ^5^ Epidemiology and Biostatistics Group, Graduate School, Universidad CES, Medellín, Colombia; ^6^ Faculty of Medicine, Universidad de los Andes, Bogotá, Colombia

**Keywords:** quality of life, European Quality of Life-5 Dimensions (EQ-5D), European Quality of Life Visual Analog Scale (EQ-VAS), Hospital Anxiety and Depression Scale (HADS), liver transplantation (LT)

## Abstract

**Background:**

Liver transplantation (LT) improves survival in end-stage liver disease. Several reports have addressed the impact of LT on patients’ lives, beyond purely medical outcomes. Although the quality of life and mental health have been demonstrated to improve with this procedure, such studies are still missing in Latin America.

**Methods:**

Patients who received LT at the Fundación Santa Fe de Bogotá between 2017 and 2019 were assessed for quality of life (QoL), anxiety, and depression and they were followed up for one year after the procedure. Pre-transplant data were gathered at inclusion on the waiting list, while post-transplant data at 3- and 12 months after LT. European Quality of Life-5 Dimensions (EQ-5D) and European Quality of Life-Visual Analog Scale (EQ-VAS) instruments were used to evaluate QoL. The Hospital Anxiety and Depression Scale (HADS) was used for evaluating anxious and depressive symptoms.

**Results:**

115 recipients met the inclusion criteria. Mean pre-transplant EQ-VAS was 70.78, rising to 87.16 and 92.56 at 3- and 12-months, respectively. Improvements in all EQ-5D dimensions were found in response to LT. According to the HADS questionnaire, anxiety was reduced by 2.35 points and depression by 1.63 points after LT.

**Conclusion:**

in the short term, LT is a successful strategy for enhancing QoL, anxiety, and depression in patients with liver disease. Long-term benefits must be assessed.

## 1 Background

Chronic liver disease causes significant physical and psychological impairments secondary to the reduction of the synthetic and detoxifying functions of the liver, as well as complications from portal hypertension and long-lasting cholestasis (1). The likelihood of surviving without a liver transplant (LT) for patients with cirrhosis depends on disease severity, which is assessed using two systems: the MELD score and the Child-Pugh score (2). For instance, 96.5% ± 0.3% of patients with a MELD score below 15 survive for 90 days, while only 15.6% ± 2.5% survive with a score of 40 (3). This contrasts with the overall survival rates of LT recipients, which reach up to 94.4% at one year and 84.1% at five years post-procedure (4). The significant trend toward excellent survival rates has prompted transplant teams to focus not only on medical outcomes but also on ensuring that transplantation enhances patients’ quality of life (QoL) (5–7).

QoL is defined as an individual's perception of his position in life, framed within the set of cultural values in which he is immersed, and that closely defines his goals, expectations, norms, and concerns. It is a broad concept that comprehensively includes the person's physical and psychological health, beliefs, social relationships, and environmental interaction (8). Health-related QoL is based on self-reported physical and mental health measures, including the ability to be socially active (social well-being) (9). The QoL of cirrhotic patients is significantly lower than that of the general population (10–13). Patients with liver disease experience a reduced QoL due to the emotional and physical burdens of the disease. Additionally, psychiatric disorders such as anxiety and depression, along with the perception of frailty, often further impact patients’ QoL (14).

The impact of the transplantation on QoL has raised recent interest in the transplant community (15). Beginning with the waiting list, patients listed for a LT typically experience worsening physical and psychological health, which is further aggravated by the uncertainty of their transplant status (16, 17). However, while Casanovas et al. identified that QoL improvement becomes evident as early as three months after transplantation (13), Girgenti et al. reported that 94% of patients who underwent liver transplant (LT) described their QoL as excellent when considering factors such as physical well-being, physical symptoms, psychological symptoms, existential well-being, and support (18). These benefits seem to also be explained by an amelioration of the anxious and depressive symptoms in these patients (19, 20). Furthermore, a systematic review indicates that the QoL five years after transplantation is comparable to that of the general population. However, it is noteworthy that this improvement primarily relies on emotional and functional benefits rather than on physical improvement (21). Supporting the long-term benefits of transplantation, a study by Domingos et al. evaluating the QoL in Brazilian patients over ten years post-LT found that, except for the mental health domain, their scores were comparable to or higher than those of the general population (22).

In this context, this study aims to evaluate the impact of LT on QoL, anxiety, and depression in patients with liver disease, comparing these outcomes to their pre-transplant baseline. It also examines how these changes interact with disease severity and psychosocial factors present at the time of surgery.

## 2 Methods

### 2.1 Design

All patients who underwent LT at the Fundación Santa Fe de Bogotá between 2017 and 2019 were screened for eligibility. A specialized psychology team followed the same operational standards to evaluate all LT candidates and their support networks at the time of their inclusion on the waiting list, and they conducted sequential follow-ups after the procedure. Only patients who were successfully followed up at three- and twelve-months post-transplantation were included. QoL along with symptoms of anxiety and depression, were retrospectively analyzed for changes. QoL surveys were conducted at 3- and 12-months post-procedure, while depression and anxiety symptoms were assessed only one year after the surgery.

### 2.2 Data collection instruments

Pre-transplant data were collected in person at the time of inclusion on the waiting list, while post-transplant data were obtained through in-person or telephone appointments. These evaluations aimed to assess patients’ understanding, beliefs, and commitment to the transplant process; identify psychosocial and emotional factors; evaluate adherence to medical treatments and healthy lifestyles; examine coping strategies; assess the quality of their support networks; and develop tailored action plans based on individual needs. The questionnaires used to assess patients’ QoL, as well as anxiety and depression, were delivered in a Spanish-validated version adapted to the Colombian context (23–27).

#### 2.2.1 European Quality of Life-5 Dimensions (EQ-5D)

Developed in 1980, the EQ-5D is an instrument that assesses health-related QoL and has demonstrated validity for liver disease and post-LT period (28). From its conception, it was designed to be delivered both in person and via phone interviews (29). The EQ-5D measures the QoL by evaluating the following dimensions: mobility, self-care, usual activities, pain/discomfort, and anxiety/depression (30–32).

#### 2.2.2 European Quality of Life Visual Analog Scale (EQ-VAS)

The EQ-VAS is a component of the EQ-5D. It is a vertical scale ranging from 0 (worst imaginable health status) to 100 (best imaginable health status), allowing patients to quantitatively assess their overall health perception (23). Its validity for assessing the QoL in several conditions has been confirmed, making its application alongside the EQ-5D, appropriate for evaluating the QoL in cirrhotic patients and LT recipients (31, 32).

#### 2.2.3 Hospital Anxiety and Depression Scale (HADS)

The HADS comprises 14 items (7 for anxiety and 7 for depression); each item is scored from 0 to 3. Scores from 0 to 7 indicate the absence of symptoms, scores from 8 to 10 suggest that the disease is probably present, whereas scores from 11 to 21 indicate the presence of anxiety or depression (25–27).

### 2.3 Statistical analysis

Categorical variables are described in terms of their frequency and percentage, and they were compared using the Chi-squared test. Continuous variables were assessed for normality using the Shapiro-Wilk test. Data exhibiting a parametric distribution were presented as the mean and standard deviation (SD), while those with a non-parametric distribution were displayed as the median and interquartile range (IQR). Depending on their distribution and whether the groups were related or independent, comparisons were made using the Wilcoxon test, Kruskal-Wallis test, and Mann-Whitney *U*-test. The level of statistical significance was set at *p* < 0.05, and analyses were conducted using Real Statistics v7.9 and SPSS v27 (IBM Corp.).

### 2.4 Ethical principles statement

This study was approved by the Fundación Santa Fe de Bogotá Corporate Committee for Ethics in Research (CCEI-15246-2023). A waiver of consent was provided as these surveys are part of the standard of care for LT recipients. Clinical data were obtained from the institution's electronic medical records, adhering to the principles of anonymity, privacy, and confidentiality. Access to the information was restricted solely to the researchers. The investigators attested that their conduct was aligned with the ethical principles of the Helsinki Declaration.

## 3 Results

**Figure 1:**
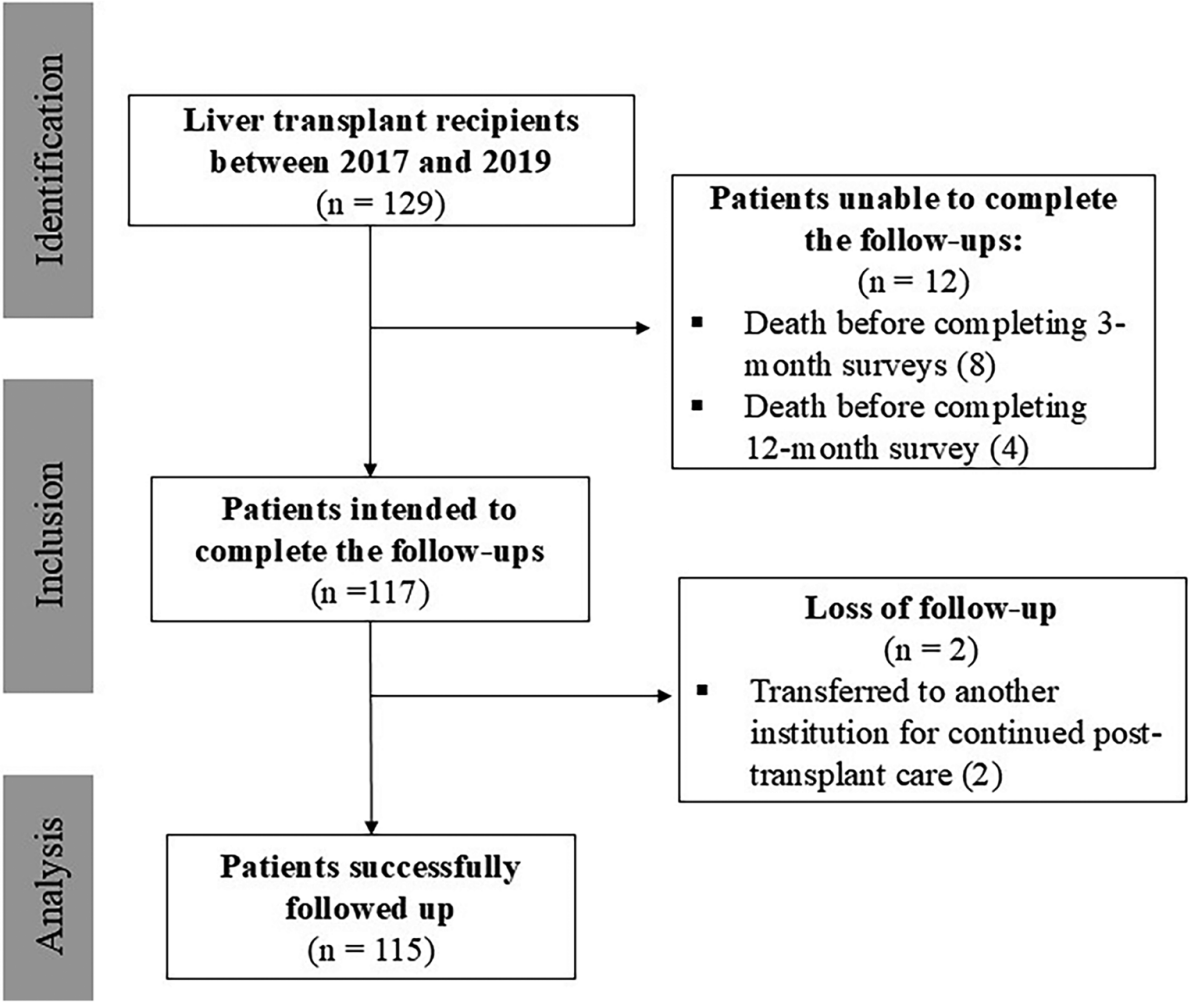
STROBE flow diagram illustrating the patient selection process.

Between 2017 and 2019, 129 patients underwent LT at our institution. Of these, 115 patients met the study criteria (Figure 1). The mean age was 58.6 ± 12 years, fifty-nine were male (51.0%), and fifty-six female (49.0%). Regarding disease severity, the majority had Child-Pugh B cirrhosis (53.0%), followed by Child-Pugh A (25.0%), and Child-Pugh C (17.0%) and 49.0% of patients had a MELD score of 15 or higher. These scores were not applied to 5 patients, as their transplants were indicated for acute hepatic failure rather than the management of cirrhosis. Autoimmune diseases were the leading cause of liver transplantation (30.4%), followed by non-alcoholic fatty liver disease (28.7%) and alcoholic hepatitis (18.3%). At the time of transplantation, 34 patients (29.6%) had a diagnosis of hepatocellular carcinoma. A comprehensive description of patient's demographic and psychosocial characteristics is shown in Table 1.

**Table 1:** Demographic and clinical characteristics.

Sex	*n*	%
Male	59	51.3%
Female	56	48.7%
Age	Mean ± SD	Min-Max
Overall	58.6 ± 12.0	16–76
Males	57.9 ± 12.6	16–75
Females	59.3 ± 11.3	24–76
Socioeconomic level	*n*	%
1	2	1.7%
2	29	25.2%
3	52	45.2%
4	26	22.6%
5	6	5.2%
Scholarship	*n*	%
None	2	1.7%
Incomplete elementary school	8	7.0%
Elementary school	11	9.6%
Incomplete baccalaureate	19	16.5%
Baccalaureate	23	20.0%
Technician	15	13.0%
Incomplete undergraduate	5	4.3%
Undergraduate	19	16.5%
Graduate	13	11.3%
Marital status	*n*	%
Marriage	48	41.74%
Free union	22	19.13%
Single	25	21.74%
Widowed	10	8.70%
Divorced	10	8.70%
Home tenancy	*n*	%
Leased	21	18.3%
Religious enclosure	1	0.9%
Family home	11	9.6%
Owned	82	71.3%
Occupation	*n*	%
Unemployed	1	0.9%
Working on profession	11	9.6%
Student	5	4.3%
Housekeeper	30	26.1%
Laid off	8	7.0%
Pensioner	21	18.3%
Employed	19	16.5%
Self-employed	20	17.4%
Child-Pugh score	*n*	%
N/A	5	4.3%
A	29	25.2%
B	61	53.0%
C	20	17.4%
MELD score	*n*	%
<15	59	51.3%
≥15	56	48.7%
Liver disease etiology	*n*	%
Alcoholic liver disease	21	18.3%
Autoimmune conditions^a^	35	30.4%
Congenital conditions^b^	5	4.3%
Pregnancy-associated acute hepatic failure	2	1.7%
Non-alcoholic fatty liver disease	33	28.7%
Secondary vascular/biliary cirrhosis	2	1.7%
Viral hepatitis	17	14.8%
Hepatocellular carcinoma	*n*	%
No	81	70.4%
Yes	34	29.6%

^a^ Including primary biliary cholangitis, primary sclerosing cholangitis, autoimmune hepatitis and cryptogenic hepatitis.

^b^ Including polycystic kidney disease, Wilson disease, Hemochromatosis and congenital hepatic fibrosis.

### 3.1 Quality of life

#### 3.1.1 EQ-VAS

**Figure 2:**
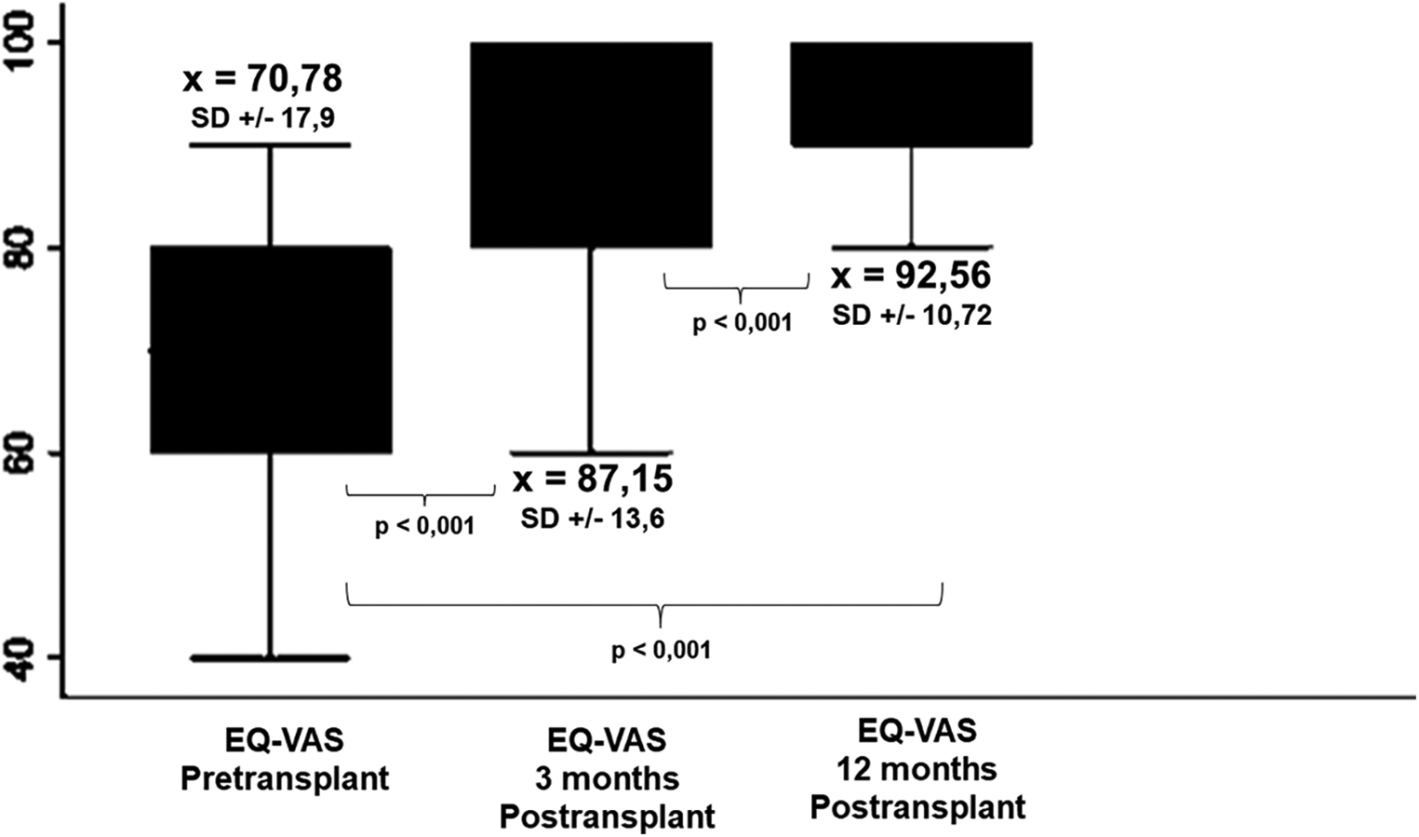
Comparison between Pre- and post-transplant EQ-VAS scores. Self-reported QoL improves as early as 3 months after transplantation and the benefit is maintained and even enhanced at 12 postoperative months.

The mean pre-LT QoL was 70.78 (SD = 17.9). It increased to 87.16 (SD = 13.61) at 3 months and to 92.56 (SD = 10.72) at 12 months post-transplant. The results compared across different time points were significant in all cases (*p* < 0.001) (Figure 2). Table 2 summarized the impact of the clinical and psychosocial variables on patients’ perception of their QoL evaluated through the EQ-VAS. None of these characteristics seems to have differentially impacted on patient's reported QoL.

**Table 2:** Effect of psychosocial and clinical variables on overall pre-transplant and 12-month post-transplant EQ-VAS.

		Pre-transplant EQ-VAS			12-month post-transplant EQ-VAS		
		Median	IQR	*p*-value	Median	IQR	*p*-value
Age >65 years		78	60–90	0.216	95	90–100	0.414
Sex	Male	70	65–80	0.330	100	90–100	0.106
	Female	70	60–80		93	90–100	
Marital status	Marriage	76	60–85	0.574	98	90–100	0.308
	Free union	70	60–90		100	90–100	
	Single	70	60–80		95	90–100	
	Widowed	65	50–75		93	90–100	
	Divorced	80	70–80		90	80–100	
Scholarship	None	50	30–(-)	0.724	100	100–100	0.489
	Incomplete elementary school	60	52.5–88.8		90	90–98.8	
	Elementary school	60	50–80		95	90–100	
	Incomplete baccalaureate	75	70–80		100	90–100	
	Baccalaureate	80	70–90		100	90–100	
	Technician	80	70–90		90	77.5–95	
	Incomplete undergraduate	50	25–77.5		85	90–100	
	Undergraduate	69	50–90		100	90–100	
	Graduate	80	67.5–80		50	30–70	
Home tenancy	Owned	70	60–80	0.340	95	90–100	0.515
	Leased	70	60–80		100	90–100	
	Family home	75	60–80		100	85–100	
	Religious enclosure	65	65–65		90	90–90	
MELD score	≥15	69	55–80	0.227	100	90–100	0.170
Child–Pugh score	N/A	50	25–75	0.052	90	82.5–97.5	0.724
	A	80	70–90			90.0–100.0	
	B	70	60–80			90.0–100.0	
	C	63	50–80			90.0–100.0	
Liver disease etiology	Alcoholic liver disease	77	60–90	0.333	100	90–100	0.426
	Autoimmune conditions^a^	75	60–80		95	90–100	
	Congenital conditions^b^	60	50–70		95	90–100	
	Pregnancy-associated acute hepatic failure	25	20–30		83	75–90	
	Non-alcoholic fatty liver disease	70	60–80		90	90–100	
	Secondary vascular/biliary cirrhosis	60	50–70		90	80–100	
	Viral hepatitis	70	60–90		100	90–100	

^a^ Including primary biliary cholangitis, primary sclerosing cholangitis, autoimmune hepatitis and cryptogenic hepatitis.

^b^ Including polycystic kidney disease, Wilson disease, Hemochromatosis and congenital hepatic fibrosis.

#### 3.1.2 EQ-5D

Table 3 illustrates patient progression in each component of the EQ-5D, from the pre-transplant evaluation to the assessments conducted at 3- and 12-months post-transplant.

*Mobility*: a significant improvement was observed in mobility between the pre-LT survey and 3- (*p* = 0.002) and 12 months post-transplant (*p* < 0.001) surveys; there was also a progressive improvement between the third and twelfth post-transplant months (*p* = 0.031).
*Self-care*: the capacity for self-care did not show significant improvement in the 3-month survey, but it did improve significantly in the 12-month post-transplant surveys compared to baseline (*p* = 0.126 and *p* = 0.043, respectively). Not significant improvement was seen between the third- and twelfth post-transplant surveys (*p* = 1.000).
*Daily activities*: patients reported an improvement when comparing pre-transplant surveys with 3- month and 12-month post-transplant surveys (*p* = 0.040 and *p* = 0.011, respectively), but no significant progression was seen between the two post-transplant questionnaires (*p* = 0.158).
*Pain and discomfort*: similarly, to the previous dimension, LT recipients reported an improvement when comparing pre-transplant surveys with both post-transplant surveys (*p* = 0.020 and *p* < 0.001, respectively), but no significant progression was seen between the two post-transplant timepoints (*p* = 0.107).
*Anxiety and depression:* the patients only demonstrated and improvement when comparing the pre-transplant surveys with the one-year post-transplant survey (*p* < 0.001). Improvement was also seen when compared the 3-month and the 12-month post-transplant reports (*p* = 0.031).

**Table 3:** Comparison between pre-transplant and post-transplant EQ-5D dimensions.

Dimension		Pre-transplant		3 months post-transplant		12 months post-transplant		Pre-transplant vs. 3 months post-transplant	Pre-transplant vs. 12 months post-transplant	3 months post-transplant vs. 12 months post-transplant
		*n*	%	*n*	%	*n*	%	*p*-value	*p*-value	*p*-value
Mobility	No problems	82	71%	104	90%	110	96%	0.002	<0.001	0.031
	Some problems	29	25%	11	10%	5	4%			
	Unable to walk	4	3%	0	0%	0	0%			
Self-care	No problems	100	87%	110	96%	114	99%	0.126	0.043	1.000
	Some problems	12	10%	5	4%	1	1%			
	Unable to wash/dress	3	3%	0	0%	0	0%			
Usual activities	No problems	71	62%	101	88%	108	94%	0.040	0.011	0.158
	Some problems	36	31%	13	11%	7	6%			
	Unable to do usual activities	8	7%	1	1%	0	0%			
Pain and discomfort	No pain/discomfort	55	48%	81	70%	93	81%	0.020	<0.001	0.107
	Some pain/discomfort	57	50%	30	26%	21	18%			
	Extreme pain/discomfort	3	3%	4	3%	1	1%			
Anxiety and depression	Not anxious/depressed	46	40%	92	80%	88	77%	0.055	<0.001	0.031
	Moderate anxious/depressed	66	57%	20	17%	27	23%			
	Extremely anxious/depressed	3	3%	3	3%	0	0%			

When analyzing each patient's progress across the dimensions (Supplementary Table S1), we found that out of the 29 patients who initially reported difficulties walking, 25 experienced symptom resolution by 12 months post-transplant (*p* = 0.031). Similarly, self-reported moderate anxiety and depression improved in 71.2% of cases, with extremely anxious or depressed patients showing significant improvement: 33.3% reported no problems, and 66.7% experienced at least moderate symptoms (*p* = 0.031). Furthermore, all 15 patients who reported self-care difficulties resolved their issues within a year post-transplant, although these differences were not statistically significant (*p* = 1.000). A comparable trend was observed in the dimension assessing usual activities, where only 11.4% of patients with reported issues and 12.5% of those unable to perform usual activities showed no change in their symptoms after one year (*p* = 0.158). Pain and discomfort also demonstrated improvement, with only 24.6% of patients reporting some pain and discomfort, and 33.3% experiencing extreme pain and discomfort, persisting at the 12-month mark. Supplementary Table S1 provides a more detailed analysis of each patient's progress across the various dimensions of the EQ-5D at different time points.

### 3.2 Anxiety and depression

#### 3.2.1 HADS

**Figure 3:**
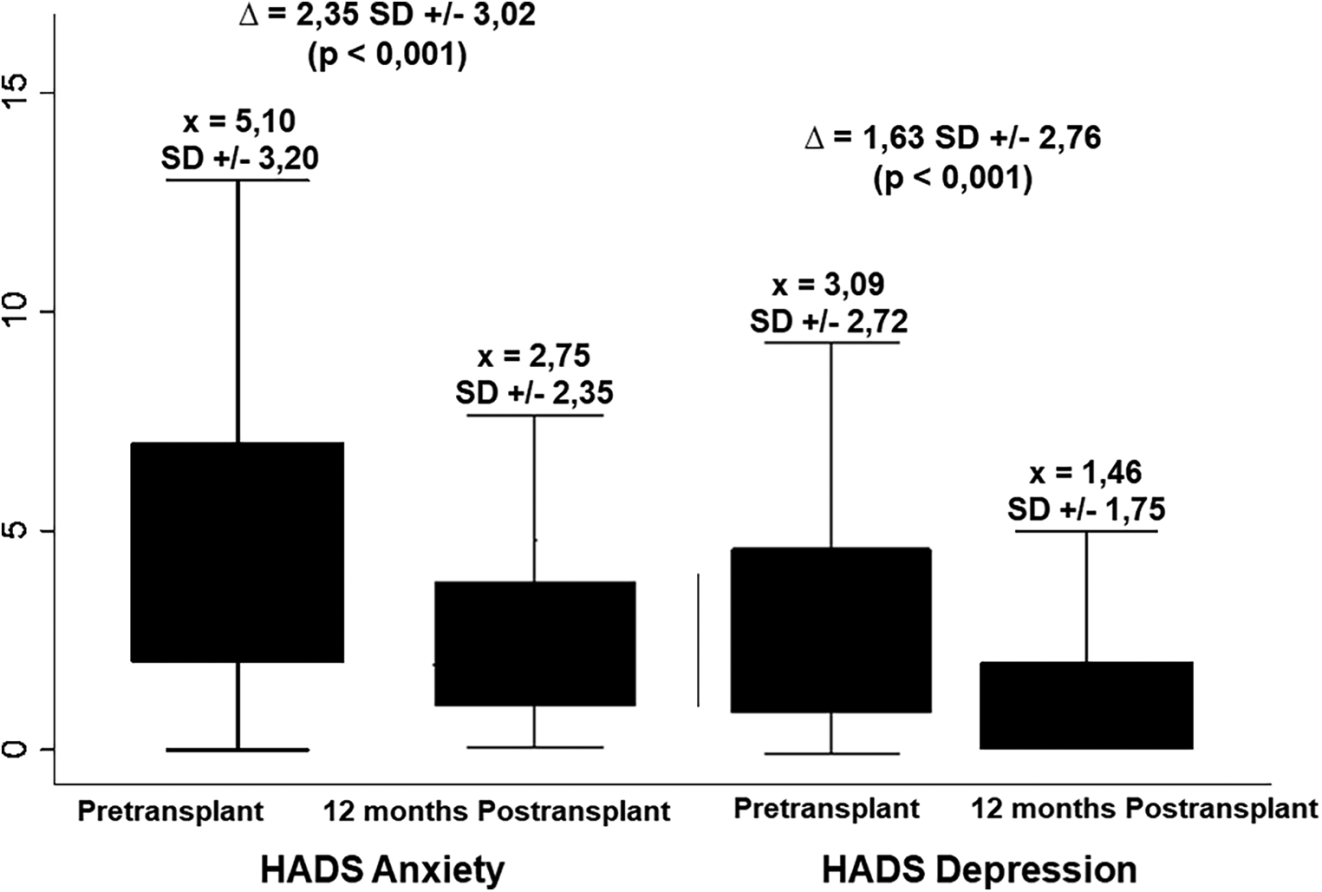
Changes in pre-transplant anxiety and depression according to the HADS tool at 12 months after LT. For both categories, a significant decrease was obtained with the LT.

Figure 3 depicts the overall anxiety and depression scores yielded by the HADS questionnaire. For anxiety, a mean difference of 2.35 ± 3.02 points was observed between the pre-transplant (5.10 ± 3.20) and post-transplant (2.75 ± 2.35) measurements. Regarding depressive symptoms, a mean difference of 1.63 ± 2.76 points was observed between the two periods (3.09 ± 2.72 and 1.46 ± 1.75, respectively). In all the scenarios the differences were statistically significant (*p* < 0.001). Table 4 displays how the clinical and psychosocial baseline characteristics interact with the overall HADS score during both pre-transplant and post-transplant stages. In this analysis, it was shown that females achieved up to three times more abnormal HADS score when compared to males during the pre-transplant stage (30.9% vs. 11.1%, *p* = 0.001), although this difference disappeared after receiving the transplant (1.8% vs. 0.9%, *p* = 0.612). Contrary to what it could be expected, patients who presented alcoholic liver disease did not have a markedly proportion of abnormal HADS scores compared to other etiologies like autoimmune conditions or the viral hepatitis (3.7% vs. 16.0% and 9.9%, respectively, *p* = 0.030). Moreover, a significant improvement from abnormal to normal scores after the transplant was seen in patients with decompensated Child-Pugh B/C liver disease.

**Table 4:** Effect of psychosocial and clinical variables on overall pre- and post-transplant HADS score.

	Pre-transplant HADS					12-month post-transplant HADS				
	Normal	%	Abnormal	%	*p*-value	Normal	%	Abnormal	%	*p*-value
	81	100.0%	34	42.0%		112	100.0%	3	2.7%	
Age >65 years	32	39.5%	12	14.8%	0.682	42	37.5%	2	1.8%	0.557
Sex										
Male	50	61.7%	9	11.1%	0.001	58	51.8%	1	0.9%	0.612
Female	31	38.3%	25	30.9%		54	48.2%	2	1.8%	
Marital status										
Marriage	36	44.4%	12	14.8%	0.116	46	41.1%	2	1.8%	0.453
Free union	13	16.0%	9	11.1%		22	19.6%	0	0.0%	
Single	20	24.7%	5	6.2%		25	22.3%	0	0.0%	
Widowed	4	4.9%	6	7.4%		10	8.9%	0	0.0%	
Divorced	8	9.9%	2	2.5%		9	8.0%	1	0.9%	
Scholarship										
None	1	1.2%	1	1.2%	0.515	2	1.8%	0	0.0%	0.559
Incomplete elementary school	6	7.4%	2	2.5%		8	7.1%	0	0.0%	
Elementary school	7	8.6%	4	4.9%		11	9.8%	0	0.0%	
Incomplete baccalaureate	10	12.3%	9	11.1%		19	17.0%	0	0.0%	
Baccalaureate	17	21.0%	6	7.4%		21	18.8%	2	1.8%	
Technician	13	16.0%	2	2.5%		15	13.4%	0	0.0%	
Incomplete undergraduate	3	3.7%	2	2.5%		5	4.5%	0	0.0%	
Undergraduate	13	16.0%	6	7.4%		19	17.0%	0	0.0%	
Graduated	11	13.6%	2	2.5%		12	10.7%	1	0.9%	
Home tenancy										
Owned	57	70.4%	25	30.9%	0.769	79	70.5%	3	2.7%	1.000
Leased	14	17.3%	7	8.6%		21	18.8%	0	0.0%	
Family home	9	11.1%	2	2.5%		11	9.8%	0	0.0%	
Religious enclosure	1	1.2%	0	0.0%		1	0.9%	0	0.0%	
MELD Score ≥15	46	56.8%	15	18.5%	0.227	59	52.7%	2	1.8%	1.000
Child-Pugh score										
N/A	3	3.7%	2	2.5%	0.352	4	3.6%	1	0.9%	0.021
A	24	29.6%	5	6.2%		27	24.1%	2	1.8%	
B	41	50.6%	20	24.7%		61	54.5%	0	0.0%	
C	13	16.0%	7	8.6%		20	17.9%	0	0.0%	
Liver disease etiology										
Alcoholic liver disease	18	22.2%	3	3.7%	0.030	21	18.8%	0	0.0%	0.433
Autoimmune conditions^a^	22	27.2%	13	16.0%		35	31.3%	0	0.0%	
Congenital conditions^b^	4	4.9%	1	1.2%		5	4.5%	0	0.0%	
Pregnancy-associated acute hepatic failure	1	1.2%	1	1.2%		2	1.8%	0	0.0%	
Non-alcoholic fatty liver disease	27	33.3%	6	7.4%		31	27.7%	2	1.8%	
Secondary vascular/biliary cirrhosis	0	0.0%	2	2.5%		2	1.8%	0	0.0%	
Viral hepatitis	9	11.1%	8	9.9%		16	14.3%	1	0.9%	

^a^ Including primary biliary cholangitis, primary sclerosing cholangitis, autoimmune hepatitis and cryptogenic hepatitis.

^b^ Including polycystic kidney disease, Wilson disease, Hemochromatosis and congenital hepatic fibrosis.

When the results are categorized as normal, borderline, and abnormal, it was observed that the percentage of patients scoring within the normal range on the HADS questionnaire increased from 43.8% in the pre-transplant stage to 80.9% in the post-transplant stage (*p* < 0.001). Notably, there was a significant improvement in the anxiety questionnaire, where the percentage of patients reporting normal levels raised from 76.5% before the liver transplantation to 95.7% afterward (*p* < 0.001) (Table 5).

**Table 5:** Overall, anxiety and depression pre-transplant and post-transplant HADS scores.

HADS Categories	Overall HADS					Anxiety HADS					Depression HADS				
	Pre-transplant		12 months after transplant			Pre-transplant		12 months after transplant			Pre-transplant		12 months after transplant		
	*n*	%	*n*	%	*p*-value	*n*	%	*n*	%	*p*-value	*n*	%	*n*	%	*p*-value
Normal (0–7)	55	43.8%	93	80.9%	<0.001	88	76.5%	110	95.7%	<0.001	108	93.9%	114	99.1%	<0.001
Borderline (8–10)	26	22.6%	19	16.5%		21	18.3%	4	3.5%		6	5.2%	1	0.9%	
Abnormal (11–21)	34	23.6%	3	2.6%		6	5.2%	1	0.9%		1	0.9%	0	0.0%	

Table 6 displayed in a more detailed way how all the individuals progressed between the two timepoints. These improvements become evident when we examine each patient's progress. For instance, among the 26 patients with a borderline overall HADS score, 73.1% achieved normal scores one-year post-transplant, while 7.7% experienced a decline. A more significant improvement was noted in the 22 patients with abnormal pre-transplant HADS scores: 64.7% moved from an abnormal to a normal score, and 32.4% improved to a borderline score, with only one patient showing no improvement (*p* = 0.001). Analyzing the HADS anxiety questionnaire reveals that all patients with abnormal scores and 81.0% of those with borderline scores achieved normal results after the transplant (*p* = 0.013). Furthermore, all patients who initially scored as borderline or abnormal on the HADS depression questionnaire reported normal scores one-year post-transplant, with only one patient who was previously normal now showing a borderline score.

**Table 6:** Patient progression in the HADS score before and after liver transplantation.

Pre-transplant		12-months post-transplant						
		Normal		Borderline		Abnormal		
		*n*	%	*n*	%	*n*	%	*p*-value
HADS overall	Normal	52	94.5%	3	5.5%	0	0.0%	0.001
	Borderline	19	73.1%	5	19.2%	2	7.7%	
	Anormal	22	64.7%	11	32.4%	1	2.9%	
HADS anxiety	Normal	87	98.9%	1	1.1%	0	0.0%	0.013
	Borderline	17	81.0%	3	14.3%	1	4.8%	
	Anormal	6	100.0%	0	0.0%	0	0.0%	
HADS depression	Normal	107	99.1%	1	0.9%	0	0.0%	(-)
	Borderline	6	100.0%	0	0.0%	0	0.0%	
	Anormal	1	100.0%	0	0.0%	0	0.0%	

## 4 Discussion

As the number of LTs increases and the survival rate improves, recent studies are focusing on the evaluation of secondary outcomes beyond purely medical results. How this procedure affects all aspects of a patient's life is a topic of high interest to the transplantation community. This study retrospectively analyzed the QoL, and the anxious and depressive symptoms in a cohort of patients undergoing LT and compared their scores with their pre-transplant baselines.

Being part of an LT program has been shown to provide benefits beyond purely medical outcomes related to liver disease; it also improves the management of other chronic conditions by ensuring timely access to various medical specialties. In Brazil, Bastos et al. found that transplantation not only significantly improved liver-related symptoms, but also other social aspects, disease-related concerns, sexual function, and sleep quality (33). In Turkey, Tamer M. and Yava A. explored the QoL in 103 LT recipients; they concluded that LT had a positive impact on recipients’ QoL and functionality, despite the need for compliance for several medical appointments that this procedure induces and the LT-related complications (34).

Our study provides insights into how LT affects QoL in a Latin American context. We found that the QoL according to the EQ-5D improves when a patient with chronic liver disease receives a LT. This benefit is kept in all the dimensions that are evaluated by this instrument. However, we found that the improvement is not evenly distributed over time, and that LT affects each dimension differently. These findings are consistent with similar studies conducted globally over the past decade (13, 15, 21, 35).

In previous reports, several demographic factors were associated with the QoL in transplant recipients, including marital status, age, gender, and occupation (15, 35, 36). Like in the general population, older patients are expected to have a decline in their QoL (37). It has also been described that LT recipients who remained employed demonstrated better QoL compared to those who were not employed (37). In terms of gender, women often report a significantly lower QoL after LT compared to men (38), although this is not always supported in all the studies (15). Regarding marital status, Mendoza-Sanchez et al. concluded that this variable does not significantly affect the QoL in LT recipients (35). In our study, we found that males and females were homogenously represented, and most were between 50 and 70 years of age. Concerning the sociodemographic characteristics, many were part of the lower-middle class according to Colombian income classification. In our setting, this is typically associated with incomplete education. Most of the patients were married, the majority owned their homes, and most were engaged in household duties. We found no significant differences in QoL across these variable categories.

The EQ-VAS analysis quantified the impact of LT on QoL. In general, a significant improvement in response to transplantation was found. We did not find any differences in this measurement between men and women during the pre-transplant period. However, at three months post-transplant, while both genders reported an enhanced QoL, women had a lower improvement compared to men. These findings are similar to those found by Cowling et al. (38). Interestingly, these differences seem to disappear twelve months after the procedure, supporting Dąbrowska-Bender et al.'s findings (15).

Interestingly, a higher MELD score did not necessarily correlate with poorer QoL, but some were observed when using the Child-Pugh score. This effect was previously described by Cristin et al. and Rabiee et al. (14, 39). These authors explain that this discrepancy happens because the MELD score does not account for symptoms associated with poorer QoL such as encephalopathy and ascites, whereas the Child Pugh score does. Importantly, our population of transplanted patients predominantly suffered from autoimmune conditions, which collectively accounted for nearly one-third of the studied cohort. This was closely followed by cases of non-alcoholic steatohepatitis. Patients with alcoholic liver disease constituted less than one-fifth of the population. This distribution differs from the patterns typically observed in other studies. The potential impact of this pattern on patient-reported QoL should be explored in future studies.

We also found a significant improvement in anxious and depressive symptoms after LT supporting the results of Mejia et al. and Martin-Rodriguez et al. (20, 35). These findings demonstrate that LT has also a positive impact on the emotional health of patients with liver disease. Of note, females had more frequently abnormal HADS scores in the pre-transplant setting similarly to what was described by Dąbrowska-Bender et al. (15), although these differences were ameliorated at twelve months post-procedure. Moreover, an inverse relationship between anxiety and depression levels and QoL was found, both at three- and twelve months post-transplant, as measured by HADS and EQ-5D. This highlights the significant role of psychological health on overall health perception beyond medical issues in LT recipients, as stated by Rabiee et al. (14).

Finally, it's important to note that instruments like the EQ-5D and the HADS primarily serve as supplementary diagnostic tools rather than definitive assessments. They should not replace a thorough psychological or psychiatric evaluation by a skilled team. Moreover, these instruments differ in their performance at detecting symptoms of anxiety and depression as the EQ-5D relies on a single self-reported question, while the HADS provides a more comprehensive evaluation of anxious and depressive symptoms.

## 5 Future perspectives

Although this and several other studies have assessed short-term QoL in LT recipients, the impact of this intervention over more extended periods remains uncertain. More studies evaluating the QoL and mental health at 3, 5, and 10 years after LT might reveal the true impact of this procedure on patients’ lives and their reintegration into daily activities. These results may also guide transplant teams to intervene in these indicators. Finally, exploring other aspects of QoL beyond those evaluated by the EQ-5D could be worthwhile. Those aspects may include sexual function and other psychosocial factors (21).

## 6 Limitations

Although QoL questionnaires have been validated for liver disease and transplantation, they are not specific to this condition, and important factors affecting QoL in these patients may have been overlooked. While there are questionnaires specifically designed for cirrhotic patients, they have yet to be validated for use in the Latin American context. Despite efforts to address several variables impacting QoL, not all could be explored, including those related to post-transplant medical conditions and medications. Therefore, confounding variables should be examined more thoroughly in future studies. Lastly, data from patients who died, were lost to follow-up, or could not be evaluated due to their medical condition were not included in the analysis.

## 7 Conclusions

The medical advancements of the last two decades have rendered LT a safe procedure for the management of chronic liver disease. Consequently, outcomes beyond mere survival must be measured to gauge the impact of transplantation on these individuals. In this context, this study demonstrated that, at least in the short term, LT is a successful strategy for enhancing the QoL of LT recipients. Our results support that in Latin America LT improves the QoL and mental health of the patients, as described in other regions. Special attention and follow-up by the medical, paramedical, and psychological teams must be ensured for women and patients displaying anxious or depressive symptoms, as these subgroups demonstrated less significant improvements in terms of QoL. Timely interventions might enhance this indicator.

## Data Availability

The raw data supporting the conclusions of this article will be made available by the authors, without undue reservation.
